# Biomechanical comparison of fixation techniques for associated both-column acetabular fractures requiring single (anterior or posterior) versus combined anterior–posterior approaches

**DOI:** 10.1007/s00402-025-06072-8

**Published:** 2025-10-24

**Authors:** Dietmar Krappinger, Huy Le Quang, Werner Schmoelz, Peter Schwendinger, Andreas E. Ellmerer, Axel Gänsslen, Richard A. Lindtner

**Affiliations:** 1 Department of Orthopaedics and Traumatology, Medical University of Innsbruck, Innsbruck, Austria; 2 Department of Trauma Surgery, Hannover Medical School, Hanover, Germany; 3 Department of Trauma and Orthopaedic Surgery, Johannes Wesling Klinikum Minden, Minden, Germany; 4 Department of Orthopaedic and Neurosurgery, Hong Ngoc–Phuc Truong Minh General Hospital, 8 Chau Van Liem, Hanoi, 100000 Vietnam

**Keywords:** Acetabular fracture, Associated both-column fracture, Fracture fixation, Posterior column screw, Anterior column screw, Biomechanics

## Abstract

**Introduction:**

Comparative data on fixation techniques for associated both-column (ABC) acetabular fractures are scarce. Compared with both column plating (AP + PP) via a combined anterior–posterior approach, single column plate plus other column lag screw fixation obviates the need for a second surgical approach. In this study, we (1) developed a clinically relevant ABC fracture model and (2) biomechanically compared the fixation strength of single column plate plus other column lag screw fixation and both column plating.

**Materials and methods:**

An ABC fracture model was created using fourth-generation composite hemipelves. Three different ABC fracture fixation techniques were biomechanically compared: (1) anterior column plate plus posterior column screw fixation (AP + PCS), posterior column plate plus anterior column screw fixation (PP + ACS), and anterior column plate plus posterior column plate fixation (AP + PP). Both single-leg stance (SLS) and sit-to-stand (STS) loading protocols were applied (loads from 50 to 750 N, ramp: 100 N/s). Fracture gap motion (FGM) and relative interfragmentary rotation (RIFR) between the four main fracture fragments were analysed at 750 N using an optical 3D measurement system.

**Results:**

Single column plate plus other column lag screw fixation techniques (AP + PCS and PP + ACS) provided comparable fixation strength to AP + PP, as evidenced by similar or even lower FGM and RIFR values. Moreover, no significant differences in FGM and RIFR were found between AP + PCS and PP + ACS. Compared to SLS loading, STS loading resulted in higher mean FGM and RIFR between the posterior iliac wing and the posterior column fragment across all fixation techniques.

**Conclusions:**

In our ABC fracture model, both column plate fixation (AP + PP) via a combined approach did not demonstrate superior biomechanical stability compared with single column plate plus other column lag screw fixation. The latter, however, requires only one approach, thereby reducing surgical time and approach-related morbidity, and appears preferable if reduction of both columns is achievable through a single approach.

## Introduction

Associated both-column (ABC) fractures are among the most common acetabular fracture types, as classified by Letournel [1, 2]. Despite their frequency, studies specifically focusing on this fracture type are limited. The available clinical studies are predominantly case series that describe distinct fixation techniques using anterior, posterior, combined, or extended approaches [3–12]. However, the lack of control groups in these studies prevents direct comparisons between fixation techniques.

Biomechanical research on ABC fractures is even scarcer. A recent literature review found that only 2 out of 36 biomechanical studies on acetabular fracture fixation addressed ABC fractures [13]. It is reasonable to assume that this scarcity is due to the heterogeneity of ABC fractures, which encompass multiple possible fracture line patterns, making it challenging to develop representative fracture models for biomechanical testing.

Nevertheless, three well-defined features are pathognomonic for ABC acetabular fractures when they occur together: (1) the eponymous involvement of both columns, (2) separation of the two columns from each other, and (3) separation of the entire acetabular articular surface from the residual iliac bone. Due to these separations, there is scientific consensus that internal fixation of both columns is necessary to allow for immediate postoperative mobilisation.

The first aim of this study was to develop a simple and reproducible biomechanical model of ABC acetabular fractures that incorporates these three key features. The second aim was to compare the fixation strength of three internal fixation strategies for ABC fractures: (1) anterior column plate plus posterior column screw fixation (AP + PCS), (2) posterior column plate plus anterior column screw fixation (PP + ACS), and (3) anterior column plate plus posterior column plate fixation (AP + PP). Biomechanical testing included both single-leg stance (SLS) and sit-to-stand (STS) loading protocols.

We hypothesised that fixation techniques requiring only a single surgical approach—either anterior (AP + PCS) or posterior (PP + ACS)—would provide comparable stability to plate fixation of both columns (AP + PP), which necessitates a combined anterior–posterior approach.

## Materials and methods

**Fig. 1:**
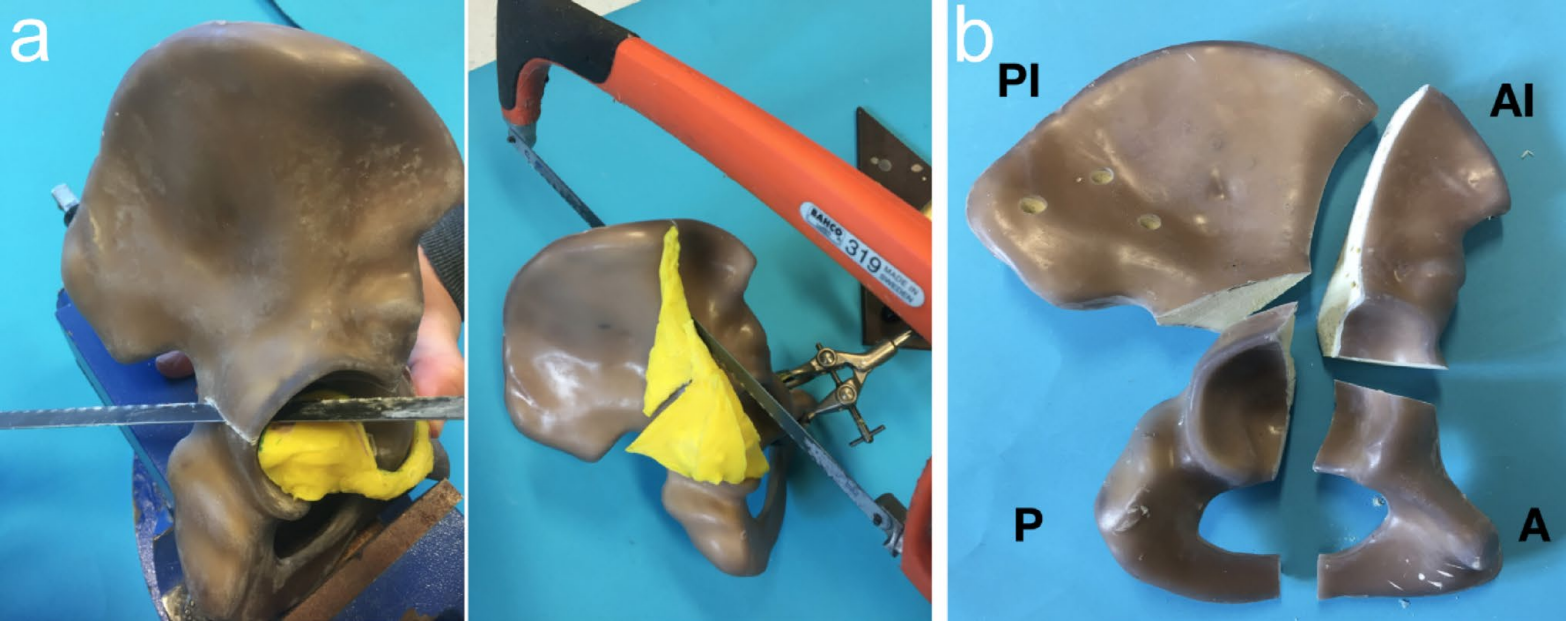
**a** Reproducible creation of the both-column fracture components using a PMMA mould, which served as a template for the osteotomy using a tenon saw. **b** The both-column fracture model and its four main fragments: (1) anterior column fragment (A), (2) anterior iliac wing fragment (AI), (3) posterior column fragment (P) and (4) posterior iliac wing fragment (PI)

We used composite hemipelves (Sawbone, Model 3409, USA) for this biomechanical study. Associated both-column (ABC) acetabular fractures were created in a reproducible manner using custom-made PMMA moulds and a tenon saw (Fig. 1a). The resulting fracture model (Fig. 1b) incorporated the typical characteristics of ABC fractures, including (1) involvement of both columns, (2) separation of the two columns from each other, and (3) separation of the entire acetabular articular surface from the residual iliac bone. As a result, the fracture model comprised the four main fragments typical of ABC fractures:

- Anterior column fragment.
- Posterior column fragment.
- Anterior iliac wing fragment.
- Posterior iliac wing fragment.

**Fig. 2:**
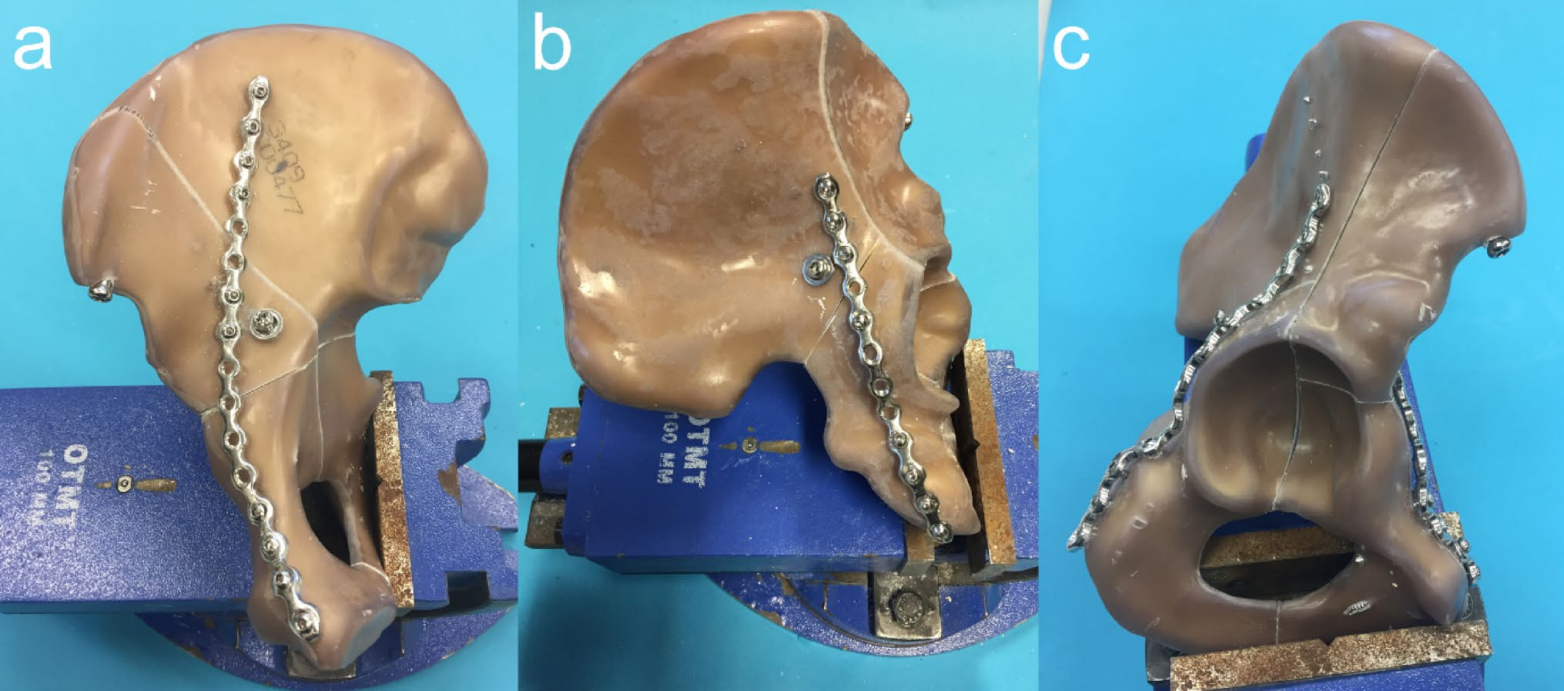
Photographs showing the 3 different fixation constructs for stabilisation of the both-column fracture: **a** anterior column plate plus posterior column screw fixation (AP + PCS), **b** posterior column plate plus anterior column screw fixation (PP + ACS), and **c** anterior column plate plus posterior column plate fixation (AP + PP). In all constructs, the fracture component between the anterior and posterior iliac wing was fixed with a partially threaded 6.5 mm cannulated screw

Fracture fixation was performed using the following implants (Fig. 2):

- Anterior plate (AP): 16-hole 3.5 mm curved low-profile stainless steel pelvic reconstruction plate with 3.5 mm cortical screws in holes 1–4, 9, 10, and 13–16.
- Posterior Plate (PP): 12-hole 3.5 mm bent straight low-profile stainless steel pelvic reconstruction plate with 3.5 mm cortical screws in holes 1–3, 5, and 9–12.
- Posterior column lag screw (PCS): Partially threaded (32 mm threads) 6.5 mm cannulated screw (90 mm length) with a predefined supracetabular entry point on the medial surface of the iliac bone (Fig. 2).
- Anterior column lag screw (ACS): Partially threaded (32 mm threads) 6.5 mm cannulated screw (90 mm length) with a predefined entry point on the posterior surface of the iliac bone.
- Iliac wing lag screw: Partially threaded (32 mm threads) 6.5 mm cannulated screw (90 mm length).

Custom-made templates were used to ensure identical implant positions for all specimens. The iliac wing screw was applied in all specimens.

The following three fixation techniques were biomechanically assessed (Fig. 2):

1. Anterior column plate combined with posterior column lag screw fixation (AP + PCS).
2. Posterior column plate combined with anterior column lag screw fixation (PP + ACS).
3. Anterior column plate combined with posterior column plate fixation (AP + PP).

The instrumented specimens were attached to the actuator of a servohydraulic material testing machine (MTS Mini-Bionix II 858; MTS, Eden Prairie, Minnesota, USA). The SI joint areas of the composite hemipelves were clamped between two steel plates with custom-made form fit moulds and securely fixed with three locking bolts. The steel plates allowed the hemipelvis to be fixed to a steel holder of the machine actuator in two different anatomical orientations. By changing the orientation of the hemipelvis, this setup enabled simulation of hip joint loading during single-leg stance (SLS, 10° anterior pelvic tilt and 0° hip flexion) and during the sit-to-stand motion (STS, 28° posterior pelvic tilt and 95° hip flexion) [14–17].

The load was applied via the head of a hemiarthroplasty prosthesis with a head diameter of 54 mm, fitting the composite hemipelves. The stem of the prosthesis itself was embedded in 9° adduction and 15° anteversion [18, 19] in epoxy resin (RenCast, Huntsman Advanced Materials, Basel, Switzerland) in a metal cylinder fixed on an x-y plane-bearing table. The loading protocol was similar to the protocol used in previous studies assessing transverse and anterior column with posterior hemitransverse acetabular fractures [16, 17]. After initially loading the 6 specimens with 5 cycles between 50 and 250 N followed by 5 cycles between 50 and 500 N with a loading frequency of 0.25 Hz for preconditioning, the specimens were loaded from 50 to 750 N (ramp 100 N/s). Six specimens were used for each fixation technique and loading protocol.

**Fig. 3:**
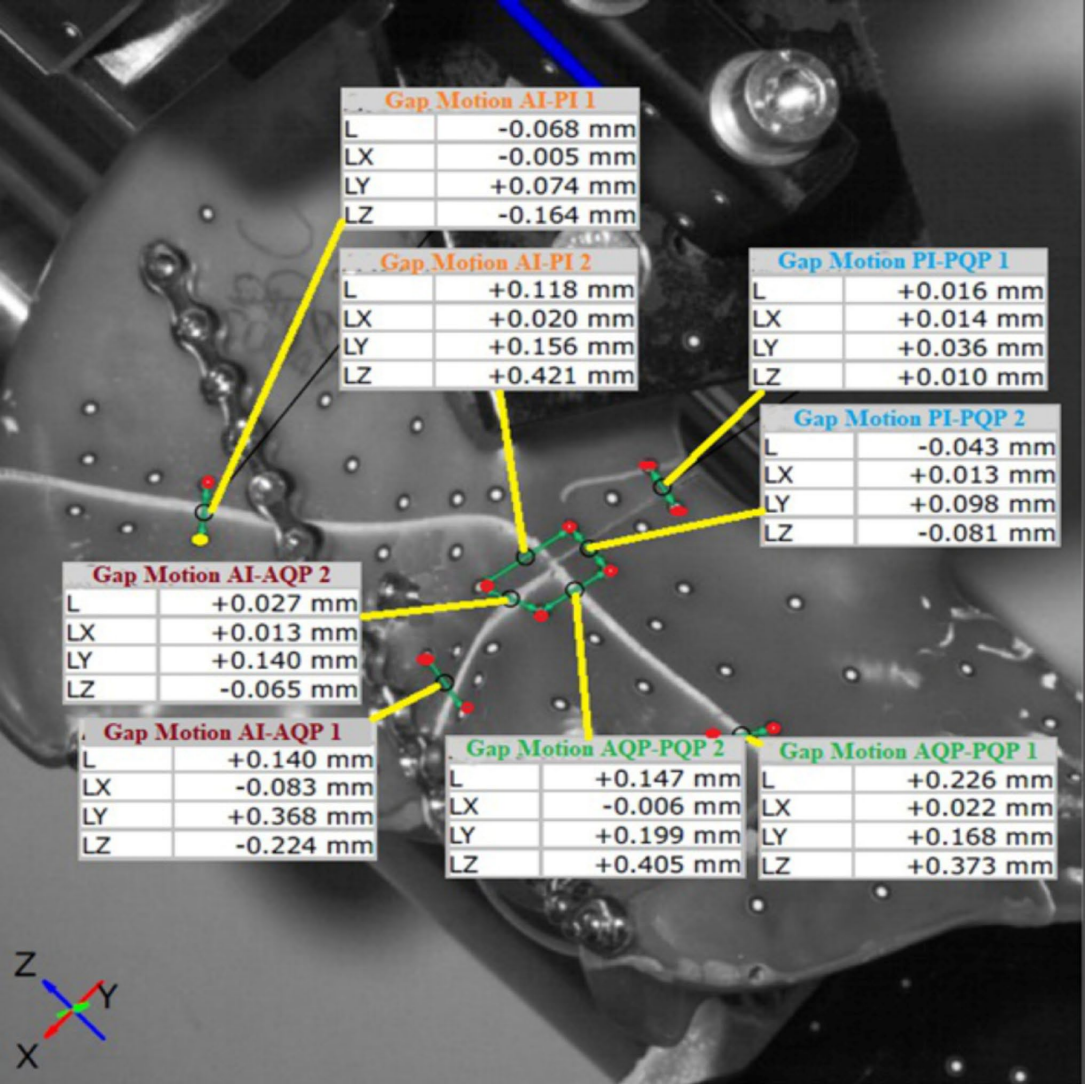
Assessment of FGM and RIFR. FGM and RIFR between the four main fragments were assessed using an optical 3D measurement system (Aramis Professional 2018, GOM). FGM and RIFR were measured at the points labelled as AQP-PQP1 (anterior column fragment - posterior column fragment), AI-PI1 (anterior iliac wing fragment - posterior iliac wing fragment), AI-AQP1 (anterior iliac wing fragment - anterior column fragment) and PI-PQP1 (posterior iliac wing fragment - posterior column fragment)

An optical 3D measurement system (Aramis Professional 2018, GOM) with passive markers was used for the measurement of fracture gap motion (FGM) and relative interfragmentary rotation (RIFR) between the abovementioned fracture fragments. FGM and RIFR were measured while loading the specimens with 750 N. For the assessment of FGM, the relative change in distance between two corresponding markers was calculated from the raw data for each point in three spatial directions (x-, y- and z-direction, Fig. 3). The resulting length of the displacement vector was then calculated using the Pythagorean theorem. The relative interfragmentary rotation (RIFR) between corresponding fragments was calculated in all three spatial axes (RIFR x-axis, RIFR y-axis and RIFR z-axis). FGM and RIFR were assessed between the anterior column fragment and the posterior column fragment (AQP-PQP1), between the anterior column fragment and the anterior iliac wing fragment (AI-AQP1), between the anterior iliac wing fragment and the posterior iliac wing fragment (AI-PI1) and between the posterior column and the posterior iliac wing fragment (PI-PQP1, Fig. 3).

SPSS Version 24.0 (SPSS, Chicago, IL, USA) was used for the statistical analysis. Data are presented as mean (minimum – maximum) value. Depending on the distribution form, a t-test for independent samples or a non-parametric Mann–Whitney test was used. For paired samples, a t-test for paired samples or a Wilcoxon test was performed. The distribution form was determined using the Kolmogorov–Smirnov test. The probability level was set at *p* < 0.05.

## Results

The results of the comparison between SLS and STS loading for all three fixation techniques are shown in Table 1. STS loading resulted in higher mean FGM and RIFR around all axes between the posterior iliac wing and the posterior column for all fixation techniques. Several of these differences were statistically significant (*p* < 0.05, marked with asterisks).

**Table 1:** Comparison of fracture gap motion (FGM) and relative interfragmentary rotation (RIFR) between single-leg stance (SLS) loading and sit-to-stand (STS) loading at 750 N (data given as mean (range))

			AP + PCS			AP + PP			PP + ACS		
			SLS	STS	*p*	SLS	STS	*p*	SLS	STS	*p*
FGM (mm)	Anterior column - posterior column		0.36 (0.16–0.80)	0.24 (0.05–0.57)	0.06	0.68 (0.40–0.98)	1.37 (0.42–2.08)	0.06	0.20 (0.10–0.32)	0.29 (0.11–0.65)	0.38
	Anterior iliac wing - posterior iliac wing		0.15 (0.07–0.24)	0.11 (0.05–0.15)	0.07	0.27 (0.10–0.48)	0.16 (0.09–0.22)	0.12	0.09 (0.03–0.15)	0.07 (0.06–0.09)	0.30
	Anterior iliac wing - anterior column		0.45 (0.18–0.86)	0.34 (0.18–0.89)	0.08	0.26 (0.10–0.41)	0.36 (0.19–0.77)	0.32	0.30 (0.21–0.49)	0.28 (0.13–0.50)	0.39
	Posterior iliac wing - posterior column		0.19 (0.07–0.27)	0.44 (0.29–0.60)	0.03*	0.29 (0.19–0.36)	1.08 (0.57–1.38)	0.00*	0.28 (0.07–0.71)	0.38 (0.12–0.82)	0.00*
RIFR (°)	Anterior column - posterior column	x-axis	0.24 (0.05–0.69)	0.21 (0.11–0.92)	0.13	0.48 (0.27–0.81)	0.63 (0.37–0.99)	0.27	0.12 (0.05–0.35)	0.52 (0.21–0.99)	0.01*
		y-axis	0.53 (0.29–0.87)	0.51 (0.19–0.85)	0.90	0.46 (0.08–1.24)	1.20 (0.52–2.04)	0.12	0.23 (0.09–0.45)	0.60 (0.15–1.60)	0.04*
		z-axis	0.57 (0.29–0.91)	0.25 (0.14–0.45)	0.06	0.53 (0.23–0.76)	1.30 (0.42–2.22)	0.06	0.33 (0.14–0.52)	0.34 (0.11–0.41)	0.91
	Anterior iliac wing - posterior iliac wing	x-axis	0.45 (0.31–0.70)	0.33 (0.18–0.56)	0.35	0.62 (0.45–0.81)	0.60 (0.42–0.91)	0.83	0.46 (0.41–0.62)	0.37 (0.19–0.62)	0.35
		y-axis	0.51 (0.24–0.74)	0.37 (0.25–0.57)	0.30	0.82 (0.51–1.07)	0.59 (0.41–0.90)	0.15	0.49 (0.24–0.76)	0.46 (0.24–0.67)	0.62
		z-axis	0.39 (0.14–0.58)	0.28 (0.10–0.38)	0.06	0.97 (0.40–1.46)	0.24 (0.06–0.36)	0.01*	0.40 (0.24–0.77)	0.14 (0.05–0.30)	0.00*
	Anterior iliac wing - anterior column	x-axis	0.64 (0.15–1.30)	0.27 (0.05–0.39)	0.04*	0.36 (0.05–1.11)	0.40 (0.08–0.56)	0.39	0.40 (0.12–0.62)	0.26 (0.08–0.50)	0.01*
		y-axis	0.42 (0.21–0.82)	0.28 (0.10–0.68)	0.01*	0.30 (0.14–0.63)	0.58 (0.18–1.51)	0.16	0.23 (0.09–0.39)	0.23 (0.12–0.46)	0.93
		z-axis	0.44 (0.26–0.67)	0.42 (0.14–0.63)	0.87	0.34 (0.09–0.69)	0.34 (0.07–0.90)	0.99	0.24 (0.12–0.40)	0.54 (0.28–1.14)	0.06
	Posterior iliac wing - posterior column	x-axis	0.52 (0.18–0.95)	0.85 (0.12–1.37)	0.14	0.24 (0.05–0.36)	1.09 (0.73–1.34)	0.00*	0.25 (0.08–0.63)	0.87 (0.43–1.18)	0.01*
		y-axis	0.49 (0.20–1.14)	0.89 (0.56–1.09)	0.03*	0.64 (0.12–1.01)	2.24 (1.48–2.71)	0.00*	0.50 (0.25–1.23)	1.12 (0.58–1.68)	0.02*
		z-axis	0.24 (0.16–0.32)	0.44 (0.23–0.82)	0.06	0.27 (0.14–0.46)	1.41 (0.86–2.23)	0.00*	0.30 (0.18–0.60)	0.46 (0.27–1.05)	0.04*

*AP + PCS* anterior column plate plus posterior column screw fixation, *AP + PP* anterior column plate plus posterior column plate fixation, *PP + ACS* posterior column plate plus anterior column screw fixation

* *p* < 0.05

The results of the comparison between AP + PCS and AP + PP for both loading protocols are shown in Table 2. Posterior column screws provided comparable, and for some parameters even higher, stability in terms of FGM and RIFR than posterior plating. Some of these differences were statistically significant (*p* < 0.05, marked with asterisks).

**Table 2:** Comparison of fracture gap motion (FGM) and relative interfragmentary rotation (RIFR) between anterior column plate plus posterior column screw fixation (AP + PCS) and anterior column plate plus posterior column plate fixation (AP + PP) (data given as mean (range))

			SLS			STS		
			AP + PCS	AP + PP	*p*	AP + PCS	AP + PP	*p*
FGM (mm)	Anterior column - posterior column		0.36 (0.16–0.80)	0.68 (0.40–0.98)	0.09	0.24 (0.05–0.57)	1.37 (0.42–2.08)	0.00*
	Anterior iliac wing - posterior iliac wing		0.15 (0.07–0.24)	0.27 (0.10–0.48)	0.10	0.11 (0.05–0.15)	0.16 (0.09–0.22)	0.08
	Anterior iliac wing - anterior column		0.45 (0.18–0.86)	0.26 (0.10–0.41)	0.11	0.34 (0.18–0.89)	0.36 (0.19–0.77)	0.88
	Posterior iliac wing - posterior column		0.19 (0.07–0.27)	0.29 (0.19–0.36)	0.96	0.44 (0.29–0.60)	1.08 (0.57–1.38)	0.01*
RIFR (°)	Anterior column - posterior column	x-axis	0.24 (0.05–0.69)	0.48 (0.27–0.81)	0.15	0.21 (0.11–0.92)	0.63 (0.37–0.99)	0.27
		y-axis	0.53 (0.29–0.87)	0.46 (0.08–1.24)	0.86	0.51 (0.19–0.85)	1.20 (0.52–2.04)	0.04*
		z-axis	0.57 (0.29–0.91)	0.53 (0.23–0.76)	0.82	0.25 (0.14–0.45)	1.30 (0.42–2.22)	0.01*
	Anterior iliac wing - posterior iliac wing	x-axis	0.45 (0.31–0.70)	0.62 (0.45–0.81)	0.16	0.33 (0.18–0.56)	0.60 (0.42–0.91)	0.03*
		y-axis	0.51 (0.24–0.74)	0.82 (0.51–1.07)	0.01*	0.37 (0.25–0.57)	0.59 (0.41–0.90)	0.08
		z-axis	0.39 (0.14–0.58)	0.97 (0.40–1.46)	0.01*	0.28 (0.10–0.38)	0.24 (0.06–0.36)	0.73
	Anterior iliac wing - anterior column	x-axis	0.64 (0.15–1.30)	0.36 (0.05–1.11)	0.45	0.27 (0.05–0.39)	0.40 (0.08–0.56)	0.72
		y-axis	0.42 (0.21–0.82)	0.30 (0.14–0.63)	0.12	0.28 (0.10–0.68)	0.58 (0.18–1.51)	0.14
		z-axis	0.44 (0.26–0.67)	0.34 (0.09–0.69)	0.12	0.42 (0.14–0.63)	0.34 (0.07–0.90)	0.62
	Posterior iliac wing - posterior column	x-axis	0.52 (0.18–0.95)	0.24 (0.05–0.36)	0.04*	0.85 (0.12–1.37)	1.09 (0.73–1.34)	0.11
		y-axis	0.49 (0.20–1.14)	0.64 (0.12–1.01)	0.31	0.89 (0.56–1.09)	2.24 (1.48–2.71)	0.00*
		z-axis	0.24 (0.16–0.32)	0.27 (0.14–0.46)	0.53	0.44 (0.23–0.82)	1.41 (0.86–2.23)	0.01*

*SLS* single-leg stance loading at 750 N, *STS* sit-to-stand loading at 750 N

* *p* < 0.05

The results of the comparison between PP + ACS and AP + PP for both loading protocols are shown in Table 3. During SLS loading, anterior column screws provided higher stability in terms of FGM and RIFR around some axes between the anterior and the posterior columns and between the anterior iliac wing fragment and the posterior iliac wing fragment (*p* < 0.05, marked with asterisks). During STS loading, this was also the case for FGM and RIFR between the posterior iliac wing and the posterior column.

**Table 3:** Comparison of fracture gap motion (FGM) and relative interfragmentary rotation (RIFR) between posterior column plate plus anterior column screw fixation (PP + ACS) and anterior column plate plus posterior column plate fixation (AP + PP) (data given as mean (range))

			SLS			STS		
			PP + ACS	AP + PP	*p*	PP + ACS	AP + PP	*p*
FGM (mm)	Anterior column - posterior column		0.20 (0.10–0.32)	0.68 (0.40–0.98)	0.00*	0.29 (0.11–0.65)	1.37 (0.42–2.08)	0.01*
	Anterior iliac wing - posterior iliac wing		0.09 (0.03–0.15)	0.27 (0.10–0.48)	0.02*	0.07 (0.06–0.09)	0.16 (0.09–0.22)	0.00*
	Anterior iliac wing - anterior column		0.30 (0.21–0.49)	0.26 (0.10–0.41)	0.62	0.28 (0.13–0.50)	0.36 (0.19–0.77)	0.47
	Posterior iliac wing - posterior column		0.28 (0.07–0.71)	0.29 (0.19–0.36)	0.94	0.38 (0.12–0.82)	1.08 (0.57–1.38)	0.00*
RIFR (°)	Anterior column - posterior column	x-axis	0.12 (0.05–0.35)	0.48 (0.27–0.81)	0.02*	0.52 (0.21–0.99)	0.63 (0.37–0.99)	0.35
		y-axis	0.23 (0.09–0.45)	0.46 (0.08–1.24)	0.26	0.60 (0.15–1.60)	1.20 (0.52–2.04)	0.09
		z-axis	0.33 (0.14–0.52)	0.53 (0.23–0.76)	0.11	0.34 (0.11–0.41)	1.30 (0.42–2.22)	0.02*
	Anterior iliac wing - posterior iliac wing	x-axis	0.46 (0.41–0.62)	0.62 (0.45–0.81)	0.07	0.37 (0.19–0.62)	0.60 (0.42–0.91)	0.01*
		y-axis	0.49 (0.24–0.76)	0.82 (0.51–1.07)	0.00*	0.46 (0.24–0.67)	0.59 (0.41–0.90)	0.06
		z-axis	0.40 (0.24–0.77)	0.97 (0.40–1.46)	0.01*	0.14 (0.05–0.30)	0.24 (0.06–0.36)	0.06
	Anterior iliac wing - anterior column	x-axis	0.40 (0.12–0.62)	0.36 (0.05–1.11)	0.67	0.26 (0.08–0.50)	0.40 (0.08–0.56)	0.64
		y-axis	0.23 (0.09–0.39)	0.30 (0.14–0.63)	0.48	0.23 (0.12–0.46)	0.58 (0.18–1.51)	0.11
		z-axis	0.24 (0.12–0.40)	0.34 (0.09–0.69)	0.49	0.54 (0.28–1.14)	0.34 (0.07–0.90)	0.34
	Posterior iliac wing - posterior column	x-axis	0.25 (0.08–0.63)	0.24 (0.05–0.36)	0.94	0.87 (0.43–1.18)	1.09 (0.73–1.34)	0.01*
		y-axis	0.50 (0.25–1.23)	0.64 (0.12–1.01)	0.31	1.12 (0.58–1.68)	2.24 (1.48–2.71)	0.01*
		z-axis	0.30 (0.18–0.60)	0.27 (0.14–0.46)	0.72	0.46 (0.27–1.05)	1.41 (0.86–2.23)	0.00*

*SLS* single-leg stance loading at 750 N; *STS* sit-to-stand loading at 750 N

* *p* < 0.05

The results of the comparison between AP + PCS and PP + ACS for both loading protocols are shown in Table 4. Both techniques provided similar results, with no significant differences between the two groups.

**Table 4:** Comparison of fracture gap motion (FGM) and relative interfragmentary rotation (RIFR) between anterior column plate plus posterior column screw fixation (AP + PCS) and posterior column plate plus anterior column screw fixation (PP + ACS) (data given as mean (range))

			SLS			STS		
			AP + PCS	PP + ACS	*p*	AP + PCS	PP + ACS	*p*
FGM (mm)	Anterior column - posterior column		0.36 (0.16–0.80)	0.20 (0.10–0.32)	0.22	0.24 (0.05–0.57)	0.29 (0.11–0.65)	0.59
	Anterior iliac wing - posterior iliac wing		0.15 (0.07–0.24)	0.09 (0.03–0.15)	0.81	0.11 (0.05–0.15)	0.07 (0.06–0.09)	0.11
	Anterior iliac wing - anterior column		0.45 (0.18–0.86)	0.30 (0.21–0.49)	0.14	0.34 (0.18–0.89)	0.28 (0.13–0.50)	0.61
	Posterior iliac wing - posterior column		0.19 (0.07–0.27)	0.28 (0.07–0.71)	0.93	0.44 (0.29–0.60)	0.38 (0.12–0.82)	0.47
RIFR (°)	Anterior column - posterior column	x-axis	0.24 (0.05–0.69)	0.12 (0.05–0.35)	0.09	0.21 (0.11–0.92)	0.52 (0.21–0.99)	0.94
		y-axis	0.53 (0.29–0.87)	0.23 (0.09–0.45)	0.07	0.51 (0.19–0.85)	0.60 (0.15–1.60)	0.74
		z-axis	0.57 (0.29–0.91)	0.33 (0.14–0.52)	0.09	0.25 (0.14–0.45)	0.34 (0.11–0.41)	0.84
	Anterior iliac wing - posterior iliac wing	x-axis	0.45 (0.31–0.70)	0.46 (0.41–0.62)	0.91	0.33 (0.18–0.56)	0.37 (0.19–0.62)	0.60
		y-axis	0.51 (0.24–0.74)	0.49 (0.24–0.76)	0.15	0.37 (0.25–0.57)	0.46 (0.24–0.67)	0.22
		z-axis	0.39 (0.14–0.58)	0.40 (0.24–0.77)	0.88	0.28 (0.10–0.38)	0.14 (0.05–0.30)	0.14
	Anterior iliac wing - anterior column	x-axis	0.64 (0.15–1.30)	0.40 (0.12–0.62)	0.12	0.27 (0.05–0.39)	0.26 (0.08–0.50)	0.95
		y-axis	0.42 (0.21–0.82)	0.23 (0.09–0.39)	0.10	0.28 (0.10–0.68)	0.23 (0.12–0.46)	0.32
		z-axis	0.44 (0.26–0.67)	0.24 (0.12–0.40)	0.13	0.42 (0.14–0.63)	0.54 (0.28–1.14)	0.52
	Posterior iliac wing - posterior column	x-axis	0.52 (0.18–0.95)	0.25 (0.08–0.63)	0.07	0.85 (0.12–1.37)	0.87 (0.43–1.18)	0.83
		y-axis	0.49 (0.20–1.14)	0.50 (0.25–1.23)	0.79	0.89 (0.56–1.09)	1.12 (0.58–1.68)	0.21
		z-axis	0.24 (0.16–0.32)	0.30 (0.18–0.60)	0.29	0.44 (0.23–0.82)	0.46 (0.27–1.05)	0.92

*SLS* single-leg stance loading at 750 N; *STS* sit-to-stand loading at 750 N

* *p* < 0.05

## Discussion

**Fig. 4:**
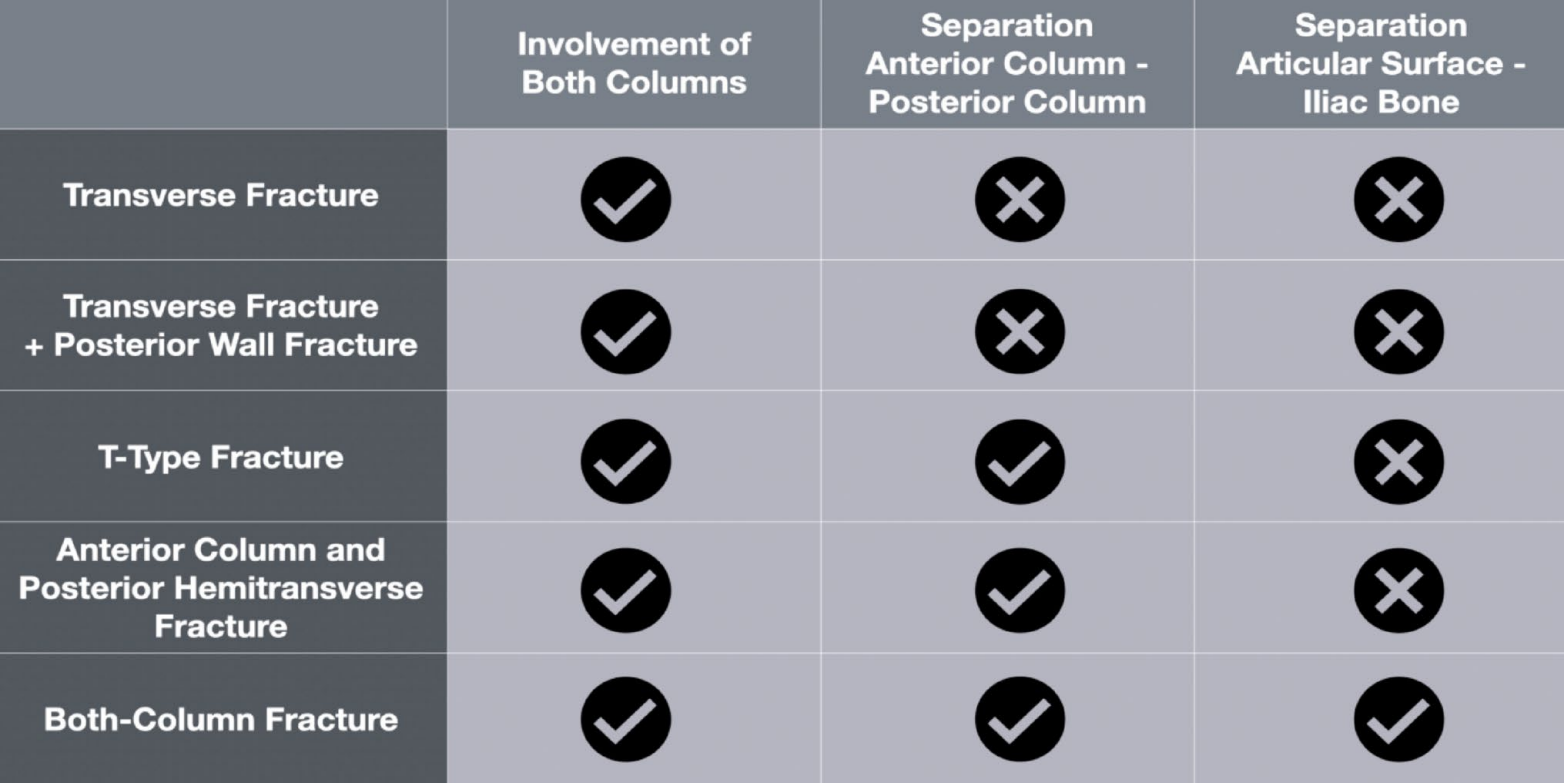
Characteristics of acetabular fractures involving both columns

Despite being among the most frequent acetabular fracture types [1, 2], associated both-column (ABC) fractures are underrepresented in the literature, with relatively few clinical and biomechanical studies specifically addressing them [13]. This underrepresentation may be attributed to the heterogeneity of ABC fractures and the somewhat misleading terminology used for this fracture type. In fact, five of the ten fracture types in the Letournel classification [1] involve fractures of both columns: pure transverse fractures, associated transverse plus posterior wall fractures, T-shaped fractures, associated anterior column plus posterior hemitransverse (ACPHT) fractures, and ABC fractures. Figure 4 illustrates a simple method to differentiate ABC fractures from other acetabular fracture types involving both columns. Transverse fractures involve both columns but lack separation between the anterior and posterior columns and between the entire acetabular articular surface and the residual iliac bone. T-type fractures as well as ACPHT fractures show separation of the anterior and posterior columns but no separation of the entire acetabular articular surface from the residual iliac bone. ABC fractures are characterised by the presence of all three key features: involvement of both columns, separation of the anterior and posterior columns, and separation of the entire acetabular articular surface from the residual iliac bone. The heterogeneity of ABC fractures is primarily due to potential additional fractures of the anterior column and the posterior wall [20]. To reduce complexity and enhance the reproducibility of our model, we focused on these three defining characteristics, excluding additional fracture lines (Fig. 1b).

Open reduction and internal fixation are considered the gold standard for the treatment of displaced acetabular fractures in non-geriatric patients [21, 22]. Acetabular fractures that involve both columns and feature separation of the anterior and posterior columns (i.e. T-shaped fractures, ACPHT fractures, and ABC fractures) are generally considered to require internal fixation of both columns to allow for immediate postoperative mobilisation [16, 23–26]. A recent biomechanical study demonstrated that internal fixation of both columns is beneficial even for simple transverse fractures [16]. Plate fixation of both columns via a combined anterior–posterior approach enables direct open reduction of both the anterior and posterior fracture components [27]. However, this technique is associated with increased approach-related morbidity, surgical time, and blood loss [28, 29]. Internal fixation of both columns via a single either anterior or posterior approach allows to reduce the overall invasiveness of this procedure. This strategy may involve the use of posterior column screws via an anterior approach or anterior column screws via a posterior approach. Yet, whether this concept provides biomechanical stability comparable to both column plate fixation in ABC fractures has remained unclear.

In our study, we identified three major findings. First, STS loading resulted in higher mean FGM and RIFR between the posterior iliac wing and the posterior column for all three fixation techniques (Table 1). This is not a surprising, as it is well-known that sitting and standing up place higher loads on the posterior structures of the acetabulum. What is more surprising is that most biomechanical studies assessing acetabular fracture fixation apply only a single-leg stance (SLS) loading protocol [30–34]. Second, our data show that both anterior and posterior column screws combined with other column plating provided similar or even higher stability in terms of FGM and RIFR than plate fixation of both columns (Tables 2 and 3). Third, no significant differences between the AP + PCS group and the PP + ACS group were found (Table 4). Therefore, plate fixation of one column combined with screw fixation of the other column via a single approach appears to be a valuable internal fixation strategy for ABC fractures from a biomechanical point of view.

However, beyond biomechanics, fracture reduction is the most important surgery-related factor determining clinical outcomes after internal fixation of displaced acetabular fractures [35–37]. Accordingly, adequate reduction of the other column is mandatory when performing internal fixation of acetabular fractures involving both columns via a single approach. Transverse fractures may be most suitable for this technique due to their single fracture line. In ACPHT fractures, the quadrilateral plate remains in continuity with the posterior column, allowing posterior column reduction via the quadrilateral plate [17]. The widespread adoption of anterior intrapelvic approaches in recent years, with their improved visualization of the quadrilateral plate, has facilitated this technique [38–41]. In ABC fractures, however, direct reduction of the other column is more challenging due to the aforementioned separations. In this context, the mechanism of „secondary congruence“ may be helpful. This concept describes the maintenance of a congruent relationship between both separated columns and the femoral head without significant articular steps, particularly in non-operative treatment of ABC acetabular fractures [42]. Gänsslen et al. referred to this as „near anatomic fragment orientation due to ligamentotaxis“ when fixing ABC fractures via an anterior approach [3]. Therefore, intraoperative reduction of the other column must be thoroughly assessed and verified using conventional fluoroscopy or, preferably, intraoperative 3D fluoroscopy or CT scanning.

Another important aspect of ABC fractures is the role of associated posterior wall fractures. These fractures should not be equated with isolated posterior wall fractures that occur after posterior hip dislocation [43, 44]. Shin et al. [20] recommended that associated posterior wall fractures in ABC acetabular fractures can be ignored surgically if the wall fragment remains attached to the acetabulum via the labrocapsular complex of the hip joint. Otherwise, fracture fixation via a Kocher-Langenbeck approach combined with an anterior column screw is advisable.

Several limitations of our study should be noted. First, we used a standardised model of ABC acetabular fractures to reduce complexity and increase reproducibility. This model did not consider additional optional fracture components of ABC fractures or articular surface impressions, particularly of the superomedial dome. Second, our biomechanical model does not account for the impact of surrounding soft tissue structures and muscles. Third, the number of specimens used in this study is limited. A larger sample size would have increased the statistical power of the analysis. Lastly, beyond the three fixation techniques applied in this study, other internal fixation options and configurations for ABC fractures have been described in the literature.

## Conclusion

Our biomechanical data show that single column plate plus other column lag screw fixation via a single approach provides stability comparable to both column plate fixation via a combined anterior–posterior approach in ABC fractures. However, achieving adequate reduction of the other column is a prerequisite for applying this technique. While secondary congruence may assist reduction, it must be thoroughly verified intraoperatively. Consequently, not all ABC fracture configurations—such as those with irreducible other column fractures or marked other column comminution—are suitable for this less invasive strategy, and conventional AP + PP fixation may be more appropriate in such cases. From a clinical perspective, AP + PCS fixation appears the most practical alternative to AP + PP, while PP + ACS may be considered when a posterior wall fracture necessitates a posterior approach.

## Data Availability

No datasets were generated or analysed during the current study.
